# State of the art in research on the gut-liver and gut-brain axis in poultry

**DOI:** 10.1186/s40104-023-00853-0

**Published:** 2023-04-11

**Authors:** Aleksandra Beldowska, Marcin Barszcz, Aleksandra Dunislawska

**Affiliations:** 1 Department of Animal Biotechnology and Genetics, Bydgoszcz University of Science and Technology, Mazowiecka 28, Bydgoszcz, 85-084 Poland; 2 Department of Animal Nutrition, The Kielanowski Institute of Animal Physiology and Nutrition, Polish Academy of Sciences, Instytucka 3, Jabłonna, 05-110 Poland

**Keywords:** Chicken, Crosstalk, Interaction, Intestines, Microbiota

## Abstract

The relationship between the intestines and their microbiota, the liver, and the neuronal system is called the gut-liver-brain axis. This relationship has been studied and observed for a relatively short time but is considered in the development of research focused on, e.g., liver diseases and intestinal dysbiosis. The role of the gut microbiota in this relationship is crucial, as it acts on poultry’s performance and feed utilization, affecting meat and egg quality. The correct composition of the intestinal microbiota makes it possible to determine the essential metabolic pathways and biological processes of the individual components of the microbiota, allowing further speculation of the role of microbial populations on internal organs such as the liver and brain in the organism. The gut microbiota forms a complex, dense axis with the autonomic and enteric nervous systems. The symbiotic relationship between the liver and gut microbiota is based on immune, metabolic and neuroendocrine regulation, and stabilization. On the other hand, the gut-brain axis is a bidirectional interaction and information transfer system between the gastrointestinal tract and the central nervous system. The following paper will discuss the current state of knowledge of the gut-liver-brain axis of poultry, including factors that may affect this complex relationship.

## Background

The gut microbiota has an essential function in the organism, including modulation of the immune response, digestion and further metabolism of nutrients, obtaining energy for the host, and shaping the feed intake and utilization level. Animal studies have shown that the intestinal microbiota is active not only in the intestines but also interacts with other organs in the digestive system, such as the liver, and organs outside, such as the brain [1]. The gut-brain axis is a bidirectional system of interactions between the gastrointestinal tract and the central nervous system (CNS). The role of the gut microbiota in this relationship is crucial because it forms a complex, dense network with the autonomic nervous system and the enteric nervous system. The intestine comprises motor neurons, sensory neurons, interneurons, and mucosa that transmit information between the CNS and the enteric nervous system (ENS). Intestinal neurons affect the microbiota physiology, absorption, secretion, blood flow, and communication. Intestinal neurons are connected to the gastrointestinal tract using primary messengers [2]. The vagus nerve is the main intermediary in communication between the CNS and the ENS. It is made of 80% afferent fibers and 20% of drainage fibers. The afferent neurons of the vagus nerve produce cholecystokinin (CCK) and serotonin (5-hydroxytryptamine, 5-HT) peptide receptors. The vagus nerve is the main component of the parasympathetic nervous system. The termination of the vagus nerve is located in the layer of the gastric and small intestine mucosa. In the mucosa of the gastrointestinal tract, there are enteroendocrine cells (EEC). They are responsible for the secretion of more than 90% serotonin in the body. In addition, EEC express receptors that react to metabolites of bacterial origin. The gut microbiota communicates with the CNS via several possible pathways [3]. The microbiota uses one of five possible pathways: the neuroanatomical pathway, the neuroendocrine pathway, i.e., the hypothalamic-pituitary-adrenal (HPA) axis, the gut microbiome metabolism pathway, and the intestinal mucosal barrier or immune system to communicate with the nervous system. Communication through the neuroendocrine and neuroimmune systems is called remote communication. The HPA axis represents the neuroendocrine system responsible for the body’s response to the stressor. The factors that cause stress in animals include stress related to the external environment, such as heat stress and stress associated with, e.g., increasing the level of pro-inflammatory cytokines. These cytokines activate corticotropin-releasing factor (CRF) in the hypothalamus, stimulating adrenocorticotropin hormone (ACTH) secretion in the pituitary gland. At the same time, ACTH releases cortisol from the adrenal glands [4].

Numerous studies have shown that the intestinal microbiota plays a crucial role in the gut-brain axis because it produces bioactive peptides, neurotransmitters, short-chain fatty acid (SCFA), intestinal hormones, and branched-chain amino acids that affect inter-organ communication [2]. These bioactive compounds are involved in transmitting signals within the gut-brain axis, simultaneously stimulating the HPA axis. In addition, peptides directly affect the immune system of the intestinal mucosa, from which signals reach the CNS [5]. The gut-brain axis as a bidirectional system includes the endocrine, nervous and immune systems, allowing the host brain to influence the gastrointestinal tract and the organism’s homeostasis [6].

Due to increased animal mortality caused by liver disease and its complications, researchers became interested in the relationship between the liver and other internal organs. In addition, the liver synthesizes and transports bile salts and antimicrobial molecules to the intestinal lumen through the bile ducts. In this way, it controls the unlimited growth of intestinal bacteria in the intestines [7]. On the other hand, intestinal microbiota produces numerous compounds that affect the liver, e.g., SCFA. Gut-liver-brain axis is a term that has been used and described recently. The increase in its popularity and the number of publications about it have been noticeable since 2016. This review aims to collect and define the primary information on the structure and function of the gut-liver-brain axis of poultry.

## Intestinal microbiota

The intestinal microbiota consists of commensal, pathogenic, and concomitant microorganisms [1]. These microorganisms include bacteria, yeast, viruses, and protozoa [8]. It has been proven that the intestinal microbiota affects the organism’s immune response, both adaptive and innate immunity [9]. The amount and composition of the microbiota vary depending on the place of colonization. *Lactobacillus* bacteria dominate the upper gastrointestinal tract in chickens while *Clostridium*, *Enterococcus*, and *Lactobacillus* are most abundant in the small intestine and ceca [10]. Such a diversity of bacteria is associated with the function of the digestive organs since gastric juices reduce the pH, which promotes colonization by lactic acid bacteria, e.g., *Lactobacillus* spp. [11]. In the ceca, the food content stays longer during the digestive process. There is also the highest concentration of SCFAs synthesized by intestinal bacteria [12]. The butyrate belonging to the SCFA is produced by the gut bacteria such as *Faecalibacterium prausnitzii*, *Clostridium* spp., and *Fusobacterium*. Moreover, *Bifidobacterium* and *Lactobacillus* bacteria have an anti-inflammatory effect and stimulate lipid metabolism in the liver, mainly by increasing the production of SCFA. These bacteria can decarboxylate essential amino acids, thereby producing amine by-products. Excess SCFA, which is not metabolized by intestinal epithelial cells, is transported through the hepatic vein to the liver, which can be incorporated as precursors to gluconeogenesis, lipogenesis, and cholesterologenesis. Acetate and propionate inhibit endogenous lipolysis [13]. SCFAs can also act as signaling molecules. They are also associated with synthesizing neuroactive molecules, including leptin, which is transported to the brain through circulation. SCFA, by regulating the appetite, reduces the adipocyte tissue of the liver. They affect the brain causing a feeling of satiety, which reduces food intake, mainly by stimulating the secretion of GLP-1 from endocrine cells. GLP-1 suppresses the appetite by stimulating the hepatic fibers of the vagus nerve. SCFAs stimulate adipocytes to synthesize and secrete leptin. Leptin is a satiety hormone that targets the neurons of the hypothalamus to increase satiety and reduce the storage of lipids in the liver [14]. The intestinal microbiota is a source of neuromediators and hormones like serotonin, catecholamine, melatonin, and histamine that directly regulate the functioning of the intestines and indirectly modulate the functions of extraintestinal organs such as the brain, kidneys, and liver [15]. The interaction between the intestinal microbiota and the host organism is bidirectional. Microorganisms shape the functioning and development of the immune system, while the host organism’s immune system shapes the composition and diversity of the microbiota in the intestines. Communication between the microbiome and parenteral organs occurs directly with the help of toll-like receptors (TLRs) and indirectly with the help of bacterial metabolites and signaling molecules [16]. A properly balanced diet rich in fiber and unsaturated fats contributes to the increase in the abundance of anti-inflammatory bacteria, which include *Bifidobacterium* and *Akkermansia*. These microorganisms are responsible for strengthening the intestinal barrier, preventing the translocation of microorganisms through the intestinal wall and the resulting endotoxemia [17]. Calefi et al. [18] showed that *Clostridium perfringens*, along with the heat stress, induced a negative behavioral response in broiler chickens and increased the expression of c-fos, the cellular proto-oncogene of the early cellular response, in the medial nucleus of the hypothalamus and the amygdala nucleus. The composition of the intestinal microbiota in broiler chickens is presented in Table 1.

**Table 1:** The composition of the microbiota in broiler chickens, considering the sections of the digestive tract and the compounds produced by the bacteria

Bacteria	Place of occurrence in the digestive system	Compounds produced by bacteria	Compounds description	Reference
*Bifidobacterium*	Caeca, crop, gizzard, esophagus, proventriculus	Vitamins K, B_1_, and B_2_, lactic acid	Lactic acid reduces pH, facilitates digestion and absorption of metabolic products, improves intestinal peristalsis, reduces cholesterol levels, and prevents diarrhea and indigestion.	[19]
*Clostridium*	Caeca, ileum	Propionate, butyrate	The ability to strongly bind propionate to Olfr78 receptors and G-protein-coupled receptor 41 (GPR41) is associated with the hypotensive effect of propionate, while Olfr78, in combination with propionate, increases blood pressure. Propionate, by interacting with the GPR43 protein present on adipose tissue cells and intestinal endocrine cells, stimulates the secretion of the intestinal hormones PYY (peptide YY) and GLP-1 (glucagon-like peptide-1), which consequently reduces the appetite and slows down the absorption of glucose.	[20, 21]
*Enterobacter*	Caeca, crop, gizzard, ileum	Histamine	The action of histamine is related to H receptors (H1-H4). It acts as a neurotransmitter and local hormone, modulates the work of the stomach, and heart work, smooths muscle contractions and circadian rhythm, and maintains body temperature.	[22]
*Escherichia coli*	Ileum	Hydrogen sulfide	It is a toxic gas secreted by the *E. coli* in the ileum. Excess H_2_S is a problem in commercial poultry houses; exposure of the hen’s internal organs to it causes immune dysregulation in hens.	[23]
*Faecalibacterium*	Caeca, large intestine	Butyrate	Its reduction contributes to a decrease in the integrity of the gastrointestinal barrier. The increase in the abundance of butyrate restores bone density and osteocyte activity and reduces inflammation in the skeletal system.	[24]
*Lactobacillus*	Caeca, crop, gizzard, esophagus, ileum, proventriculus	Acetylcholine, serotonin, dopamine, gamma-aminobutyric acid (GABA)	Vagus nerve GABA receptor expression and hyperpolarization of enteric nervous system neurons affect pain sensation by the nervous system. Serotonin, as a neurotransmitter and tissue hormone, participates in the transmission of impulses between cells of the nervous system; it affects emotional states, concentration, and memory.	[25]

## Intestinal mucosa

The mucous membrane comprises lamina propria, epithelium, and smooth muscles. The outer layer of the small intestine is lined with absorbent cylindrical cells (enterocytes) alternately with goblet cells and enteroendocrine cells [26]. Goblet cells (GCs) are polarized epithelial cells that secrete mucins, the main constituents of mucus. Intestinal mucus is the host’s first line of defense. It protects the surface of the epithelium from pathogens, enzymes, and mechanical damage occurring during the digestion process [27]. Bacteria and components produced by goblet cells are recognized by the sensory system of the immune and intestinal cells. The intestinal mucosa is densely colonized by microorganisms capable of metabolic activity [28]. Intestinal mucus should be a barrier, catching and immobilizing pathogens while allowing nutrients to penetrate the surface of the epithelium. The compact inner layer prevents the penetration of pathogenic bacteria such as *Clostridium perfringens* and *Escherichia coli* into intestinal epithelial cells [29]. More than 90% of nutrient absorption occurs in the small intestine, with a thinner layer of mucus. In contrast, a thicker layer of mucus is found in the large intestine, preventing excessive bacterial colonization [27, 30]. Mucin is a major component of cytoplasmic granules produced by goblet cells [31]. Movements of the cytoskeleton are regulated by constitutive secretion, moving secretory granules toward the cell surface. This constant release results in the maintenance of the mucus layer. It has been observed that bacteria destroying the mucosal surface stimulate the more rapid release of stored mucin granules. The physiological dynamics of mucins are influenced by bacterial metabolites such as SCFA, lactic acid, secondary bile acids, and ammonia, which regulate immunity and intestinal mucosa physiology [32–35]. The absence of these gut bacteria in uninfected chickens reduces the number of cup cells and decreases *MUC2* gene expression [36]. The rapid production of mucins in the intestinal villi facilitates digestion and protects the villi surface from microbial invasion [32]. The sulfate group protects mucins from degradation by bacterial enzymes in the gut. Sulfated mucins reduce the ability of the pathogenic bacterium *Campylobacter jejuni* to penetrate the intestinal mucus in poultry [37]. The inner layer of the mucosa contains transmembrane mucins, while the outer layer contains secretory mucins [27]. Prebiotic galactooligosaccharides (GOS) delivered *in ovo* increase *MUC* gene expression [32] and also stimulates the growth of intestinal villi and, thus goblet cells [38]. Alemka et al. [39] demonstrated the cytotoxic effects of mucins against *Salmonella* bacteria [39]. Intestinal dysbiosis disrupts the expression of the *MUC2* gene. Oxidative stress caused by a high-fat diet increased cytokines IL-1β and IL-17 and simultaneously decreased MUC expression. The intestinal bacterium *F. prausnitzii* is an important butyrate producer with anti-inflammatory properties, while *Akkermansia muciniphila* degrades mucin in the intestinal lining, causing syntrophic interactions and stimulation of intestinal metabolites. Co-cultures of *A. muciniphila* with butyrate-producing bacteria result in syntrophic growth. Butyrate affects glucose and energy homeostasis by activating intestinal gluconeogenesis [40]. It also stimulates mucus secretion [36, 41], thus its impaired metabolism in the colon epithelium may result in a thinner adherent mucus layer [42]. One factor that may inhibit butyrate metabolism is hydrogen sulfide produced by the intestinal microbiota during the catabolism of sulfur amino acids. Hydrogen sulfide may also damage disulfide bridges of mucins, contributing to the intestinal barrier’s impairment [43].

## Gut-brain axis

The brain and intestines participate in bidirectional communication with the help of the endocrine and nervous systems. This connection has been called the gut-brain axis. Bacterial metabolites and host hormones such as leptin and glucagon-like peptide 1 and 2 regulate host metabolic homeostasis, development, health, and behavior. Changes in gut microbiota composition can affect gut health and brain changes, such as altering monoamine concentrations in crucial brain areas, i.e., decreases in norepinephrine (NE), epinephrine (E), and 5-HT in the hypothalamus and dopamine in the midbrain [44]. It has been observed that any intestinal infection activates the midbrain serotonergic system by increasing levels of 5-HT and 5-hydroxyindoleacetic acid (5-HIAA) in the hypothalamus. Accordingly, reduced levels of monoamines increase the abundance of pathogenic bacteria such as *Escherichia coli*, *Clostridium perfringens*, and *Salmonella* spp. in hens [44]. Intestinal commensal bacteria participate in the metabolism of undigested food residues, from which the body draws additional energy. The degradation of protein and carbohydrates also produces neuroactive components [11]. Neuroactive molecules include, for example, serotonin, which exerts a local influence on the regulation of physiological processes. Adequate serotonin levels positively affect gastrointestinal motility by increasing small intestinal peristalsis and decreasing gastric hydrochloric acid secretion. Decreased brain serotonin levels and increased catecholamine levels are found in animals fed only diets low in tryptophan and rich in tyrosine. The deficiency of serotonin causes a lack of appetite and aggression seizures. On the other hand, excess serotonin increases body temperature [45].

**Fig. 1:**
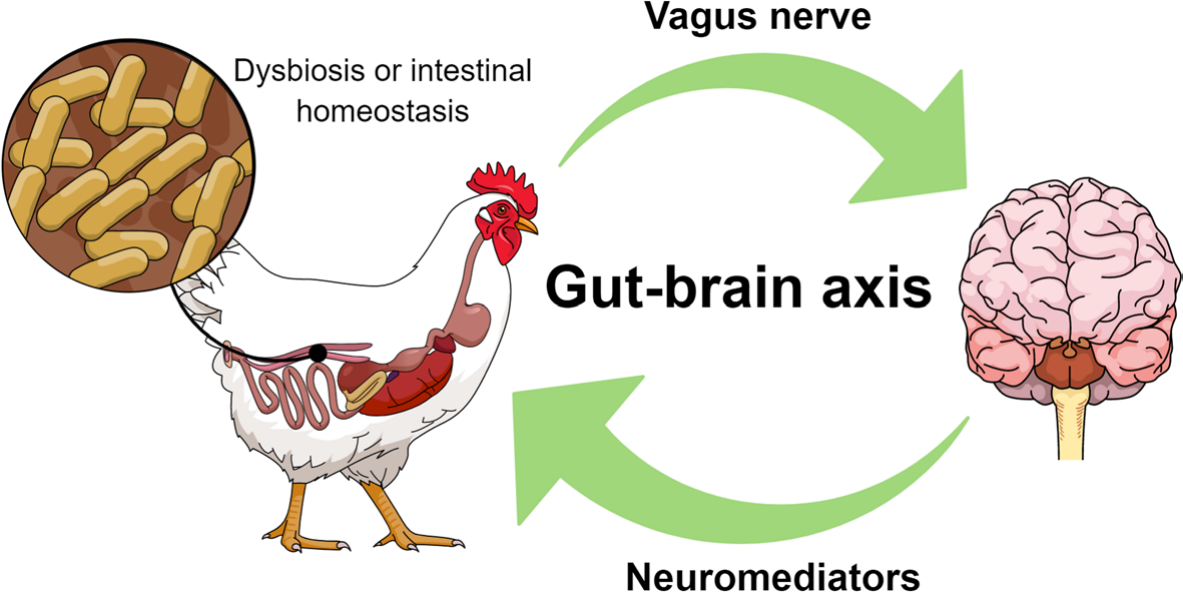
The mechanism of communication of the gut-brain axis

One neuronal pathway transmits information to the CNS via the vagus nerve afferent fibers, which can shape host behavior. The structural components of bacterial cell walls and products of bacterial metabolism are responsible for the host’s immune response by activating enteroendocrine cells that affect the nervous system locally and systemically [46]. The intestinal nervous system is the main connection between the intestinal microbiota and the host organism. It is the second most complex nervous circuit in the organism. Through neural networks and neurotransmitters, it closely connects to the CNS. Because of the number of neurons in the enteric nervous system, it is called the second brain [47]. The brain affects gut physiology, microbiota composition, and the immune system. Neurotransmitters, including NE and E, stimulate the growth of beneficial microbial bacteria such as *Lactobacillus* spp. or *Bifidobacterium* spp. [48]. Through communication with the brain, the intestinal nervous system affects, among others, the cerebral cortex, amygdaloid body, or hypocampus. These structures are responsible for the organism’s memory processes [49]. An effect of the microbiome-gut-brain axis on social behavior among animals was observed. Disturbance of the composition of microorganisms and the state of intestinal dysbiosis increases the sense of danger and can cause animals to separate from other individuals [50]. Hill et al. [51] observed the influence of the gut-brain relationship and the state of satiety in animals. Several compounds with antagonistic functions mediate endocrine regulation of appetite, i.e., those that increase food intake: neuropeptide Y (NPY) ghrelin and orexins A and B, and those associated with reduced hunger such as leptin, cholecystokinin, glucagon-like peptide-1 (GLP-1), and pancreatic peptides PP (pancreatic polypeptide) and peptide YY (PYY). Ghrelin and leptin are two compounds with opposing activities concerning energy balance and appetite regulation. The integration of signals that regulate appetite occurs in the arcuate nucleus of the hypothalamus. Groups of neurons are responsible for processing sent impulses into neural and behavioral responses. These consist of initiating a new meal when hunger appears or stopping further eating when satiety appears. The arcuate nucleus contains two antagonistically acting systems. The orexigenic system first includes orexin A and B and NPY neurons, which express appetite-stimulating substances. These substances reduce energy expenditure under starvation conditions and stimulate food intake. The second system is the anorexigenic system consisting of proopiomelanocortin (POMC) and α-melanotropic hormone (MSH). These substances decrease appetite and energy expenditure [51]. Bacterial components and metabolites stimulate the satiety pathway by stimulating endocrine cells after feed intake. Bacterial peptides like glucagon-like peptide-1 (GLP-1) and glucagon-like peptide 2 (GLP-2) act directly in the hypothalamus. They are responsible for the long-term regulation of appetite [52]. A study conducted on germ-free Japanese quails found that the state of intestinal dysbiosis reduces the emotional response to fear and social difficulties without much impact on animal growth [53]. Gentle pecking of feathers in chickens is considered normal social behavior, while strong pecking is already considered a manifestation of aggression [54]. This harmful behavior was linked to the gut-brain axis because the lines of aggressive and non-aggressive chickens exhibited a variable gut microbiome. Pecking feathers can lead to increased stimulation of the intestinal wall, thereby impairing serotonin signaling [55]. Intestinal pathogens usually cause disease states in the host organism. Such symptoms are generally accompanied by a decrease in feed intake, as a consequence of which the growth of animals is slowed down. To reduce the losses associated with the reduction of feed intake, Bacillus spores may be given to animals because they increase the frequency and duration of feeding [56]. Giving chickens tryptophan modifies the intestinal microbiota, reducing serum serotonin and heat shock protein levels. The metabolism of tryptophan to serotonin has been linked to feather pecking in birds, and its supplementation reduces this behavior [54]. Giving pro or prebiotics as a feed additive is the most practical way to shape the intestinal microbiota. In the case of poultry, these additives can also be given *in ovo* on d 12 of incubation [54]. Since many breeding situations can cause stress during rearing, and this condition affects the microbiome, it is important to take care of the proper microbiome. Bacteria synthesize neurotransmitters, including 5-HT, which acts locally. In the small intestine, 5-HT is released into the mucous membrane, affecting intestinal peristalsis, stimulating bicarbonate secretion during digestion, and dilating blood vessels [57]. In controlled conditions, microorganisms of the gastrointestinal tract play beneficial roles. They participate in competing with pathogenic bacteria and keeping the structure of intestinal mucus intact [27]. In addition, they ferment undigested polysaccharides into SCFA and provide vitamins, especially from group B [58]. The gut microbiota is a source of peripheral hormones and neurotransmitters, such as 5-HT and dopamine. These molecules directly communicate the state of intestinal health through the vagus nerve fibers, up to the brainstem and other areas of the brain. Stressful signals through peripheral and central pathways activate the hypothalamus-pituitary-adrenal axis (HPA). This can alter the composition and function of the gut microbiota, as well as the function of the intestinal epithelium [44]. The release of corticotropin-secreting factor from the hypothalamus stimulates the release of adrenocorticotropic hormone from the anterior pituitary lobe into the circulatory system. This causes the secretion of corticosteroids from the adrenal glands, such as corticosterone, in birds. Corticosteroids affect the gastrointestinal tract through direct interactions with gut cells and bacteria, thus leading to the release of cytokines (interleukin-6), which affect the brain and regulate appetite, mood, and cognitive function [59]. Regulation of appetite occurs in the hypothalamus and brainstem. Intestinal nutrients stimulate the secretion of cholecystokinin, the hormone responsible for satiety [60]. Synapses have been discovered in enteroendocrine cells, which at the same time are cells of the sensory epithelium of the intestine. They transmit sensory signals from the intestinal environment to the brain [61]. A simplified diagram of the mechanism of interaction between the microbiota and the brain is presented in Fig. 1.

## Gut-liver axis

The term gut-liver axis describes the close functional-anatomical relationship between the liver and the gut [62]. Owing to the numerous occurrences of liver diseases, interest began to be taken in the relationship between the intestinal microbiota and the liver. Intestines and the liver communicate via the portal vein and systemic circulation. Metabolites produced by the intestines are transported through the portal vein to the liver. At the same time, the liver transports bile salts and antimicrobial molecules to the intestinal lumen through the bile ducts. In this way, it controls the unlimited growth of bacteria. Antimicrobial peptides and molecules (AMPs) are a diverse class of naturally occurring molecules. They are produced as the first line of defense by all multicellular organisms. These proteins can exhibit broad activity to neutralize fungi, bacteria, yeast, viruses, and cancer cells. Antimicrobial proteins include interferon: alpha (α), beta (β), and gamma (γ) [7]. A diseased liver cannot properly inhibit bacterial overgrowth, eliminate harmful by-products, and accelerate regeneration. The liver is also one of the immune organs, activating adaptive and innate immunity mechanisms after exposure to intestinal bacteria in the circulatory system [63]. The liver is an organ with immune properties. One of the liver functions is phagocytosis, the engulfment of particles originating in tissues or entering the body from outside, primarily with blood from the portal vein. These particles are degraded cell fragments, denatured proteins, lipoproteins, viruses, bacteria, and fungi. These particles are degraded in the liver macrophages and the Kupffer cells. Kupffer cells are sedimented macrophages found between endothelial cells in the wall of sinusoidal vessels in the liver. Their main functions include participation in the body’s immune mechanisms through phagocytosis of bacteria and phagocytosis of cancer cells [63]. In addition, the liver is the main site of plasma protein synthesis. Hepatocytes, the liver cells, are responsible for the secretion of immunoglobulins, albumin, and fibrinogen. Hence, liver dysfunction usually decreases plasma protein production [64]. The enterohepatic axis represents a close bidirectional relationship between the intestine and the liver. The liver is an organ exposed to the products of digestion and absorption, in addition to all factors coming from the intestines, which include bacteria and components of bacterial origin, e.g., lipopolysaccharides (LPS), SCFAs, ammonia, phenols, toxins, and carcinogens previously neutralized in the liver, which are re-released by the bacteria and end up in the gut-liver circulation. The liver produces bile which is later stored in the gallbladder. Bile is composed of emulsifying fats, bile salts, and bile pigments. These include bilirubin, which is a breakdown product of hemoglobin. The small intestine uses liver bile to break down fats. In addition, the liver stores glycogen, buffers blood glucose levels and thus participates in carbohydrate metabolism. Excess sugars in the liver are converted into fatty acids. The liver is also involved in the breakdown of amino acids, a process in which liver cells convert a toxic by-product (ammonia) into urea [65].

The venous system of the portal circulation determines the enterohepatic axis and emphasizes the importance of anatomical and functional interaction of the gastrointestinal tract and liver [66]. The portal vein is a direct venous outflow from the intestine. Increased intestinal barrier permeability automatically exposes the liver to numerous toxic components of intestinal origin, including intestinal bacteria such as *Escherichia coli* [67]. Intestinal dysbiosis is associated with increased intestinal permeability and, consequently, with exposure of the liver to bacterial components. These are called molecular patterns and are divided into two groups, pathogen-associated molecular patterns (PAMP) and damage-associated molecular patterns (DAMP). Both patterns can cause liver damage [68]. Pathogen-specific patterns (PSPs) act directly on hepatocytes and/or cells of the liver’s innate immune system, including Kupffer cells and starlit cells. The activated immune system of the liver stimulates pro-inflammatory, antiviral, and antiapoptotic pathways in hepatocytes. Such a reaction has both positive and negative effects. The harmful effect is the activation of the immune response and the release of pro-inflammatory cytokines, while the positive effects include hepatocyte reconstruction and cytoprotection. The liver, through bile acids and secretory immunoglobulin A (sIgA) affects the intestinal microbiota, regulating the microbial population. Immunoglobulin synthesis in the gut is one of the body’s initial protective responses to pathogenic bacteria in the intestinal contents. Bile produced in the liver is the main source of sIgA in the intestinal lumen. sIga’s primary function is to prevent pathogenic bacteria and viruses from attaching to and attacking erythrocytes. The concentration of immunoglobulins in the gut depends on the amount of sIgA in the gastrointestinal tract and can vary from day to day [69, 70]. In the future, therapies are predicted in which it will be possible to use an artificial microbiota to reduce the permeability of the intestinal barrier and reduce the release of pro-inflammatory cytokines in the intestine. Disturbed integrity of the intestinal barrier caused by dysbiosis leads to an increase in bacterial translocation and metabolic endotoxemia, which activates the hepatic TLR system, thus the local inflammatory response of the liver [71]. Bacterial metabolites, which include SCFAs and bile acids, are heavily involved in normal liver function and reduced lipogenesis and liver inflammation. Aberrations occurring in the composition, diversity, and function of the commensal microbiome lead to increased intestinal permeability, LPS production, ethanol production, and bile production. All these metabolites and factors combined with lipids from food can cause liver disorders. These include steatosis, inflammation, and liver damage. Liver disorders such as primary cholangitis or spotted liver disease in poultry farming can be caused by both qualitative and quantitative changes in the gut microbiota [72, 73]. A healthy liver is a barrier between systemic circulation and the intestines. In the case of disturbances in the functioning of the liver, this barrier is dysfunctional. Links were found between liver disease and the composition of the microbiome.

**Fig. 2:**
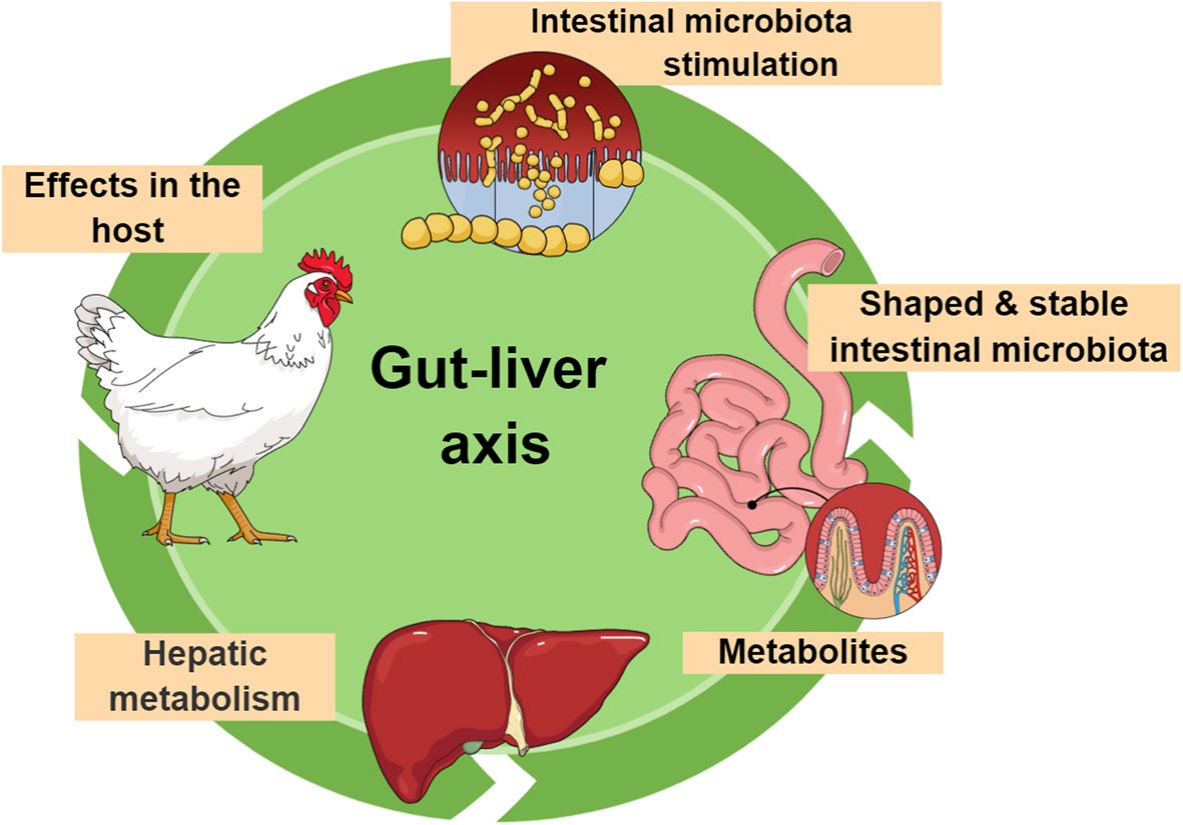
Simplified diagram of the gut-liver axis mechanism in chicken

The diagram of the relationship between the microbiota and the gut is presented in the Fig. 2.

## Factors affecting the gut-liver-brain axis in poultry

### Nutrients

Nutrients significantly affect the brain, liver, and most internal organs by affecting their development and functioning during health and disease. Diet is the most important modulator of the intestinal microbiota, both in terms of its development and biosynthetic abilities. Amino acids are one of the essential nutrients supplied to the host by the intestinal microbiota. They act as neurotransmitters (*L*-glutamate). They can also be precursors for the synthesis of neurochemicals, including serotonin, gamma-aminobutyric acid (GABA), dopamine, and norepinephrine. Strains of *Lactobacillus* and *Bifidobacterium* bacteria can metabolize amino acids [74]. This is beneficial in the context of animals, where intestinal microorganisms provide amino acids that are unavailable in their daily diet. Amino acids, the end product of protein digestion, are absorbed into the blood vessels of the intestinal villi and transported to the liver through the portal vein. Amino acids act as precursors and signal the animal’s nutritional status to the brain [74]. Excessive consumption of sugars affects changes in the brain’s functioning and its impairment. It has been mainly observed that sugars reduce animal learning ability and memory [75].

Excessive fiber consumption stimulates the abundance of *Bacteroides thetaiotaomicron*, which increases the amount of free sialic acid. Such a reaction may contribute to the growth of the pathogenic bacterium *Clostridium difficile* [76]. Dysregulation of SCFA interferes with metabolism and sleep [77]. In turn, omega-3 deficiency increases aggression and arousal [78]. Dietary fiber increases the ratio of Firmicutes to Bacteroidetes, while the ketogenic diet causes the growth of *Akkermansia*, which modulates host amino acids’ metabolism [79]. The diet-microbiome interaction is based on the action of metabolites and nutritional components derived from the diet on the host organ systems. Salah et al. [80] experimented on chicken broilers exposed to heat stress (8 h, 34 °C) and fed a diet supplemented with curcumin (100 mg/kg diet). These studies showed that the addition of curcumin to a diet of heat stressed-chickens doubled the level of coenzyme Q10 in the liver [80].

Additionally, curcumin reduced the influence of thermal stress on the level of the enzyme Na/K ATPase in the liver. The addition of curcumin reduced the percentage of unwanted fat deposits in the abdominal cavity in heat-stressed broilers, which may be due to its soothing effect on energy metabolism. Curcumin increased serotonin levels in the brain of broilers to the level of the control group. This study shows that nutrients are crucial in the brain’s functioning, and undisturbed animal behavior [80].

Nutrition and compounds produced by bacteria can also affect up and down-regulation of gene and protein expression. Downward regulation is when a cell reduces the amount of a cellular component, such as RNA or protein, in response to external stimuli. An example is a cellular decrease in receptor expression in response to its increased activation by hormones or neurotransmitters. This results in a decreased sensitivity to the molecule. During downward regulation, intestinal cells produce signaling molecules that circulate in the blood and pass through the blood-brain barrier to the CNS. Salt in the diet stimulates the response of Th17 cells in the intestine, which induces an increased amount of interleukin-17 in the plasma. IL-17 affects the endothelial cells of the brain and inhibits the production of nitric oxide, thereby reducing brain perfusion [81]. Upward regulation involves the response of liver cells exposed to xenobiotic molecules. This increases the degradation of such molecules. Up-down adjustment is carried out thanks to operating three systems: autonomic nervous system (ANS), enteric nervous system (ENS), and central nervous system (CNS). ANS refers to the sympathetic and parasympathetic nerves, which controls the motility of the gastrointestinal tract and regulates blood flow in the digestive tract and the secretion of digestive juices. ANS also includes mast cells (mastocytes), found in the most outstanding amounts in blood vessels and around the endings of nerve fibers. Mastocyte granules are rich in histamine and heparin, stimulating the secretion of prostaglandins and cytokines. Their function is to receive and transmit signals to the nervous system. Their role in the mechanisms of the acquired immune response is based on the ability to present the antigen and direct the action of the released cytokines and other humoral factors [82]. ENS reacts to gastrointestinal microorganisms and converts chemical signals from the environment into nerve impulses, which are then spread to the intestines and other organs [83]. The CNS regulates the sympathetic and parasympathetic nerves, affecting the digestive system [84].

### Probiotics and prebiotics

Probiotics are cultures of living microorganisms used as functional components to shape and maintain the proper state of health of the body. They act on the intestinal microbiota to increase the activity of digestive enzymes, reduce pathogen development, and stimulate the immune system [85]. Probiotics work properly only when they survive in the gastrointestinal tract. The main probiotic bacteria are *Lactobacillus* and *Bifidobacterium* [86]. Probiotics produce lactic acid or SCFA. Studies conducted on poultry provided information on the regulating effect of *Bifidobacterium infantis* on excessive stress response through the hypothalamic-pituitary-adrenal axis [87]. In addition, numerous studies have shown that probiotics reduce the negative effects of stress. The gut microbiota can influence the central nervous system via the gut nervous system and the immune system under stress. This is due to the fact that stress increases intestinal permeability. This allows commensal microorganisms to translocate through the intestinal mucosa and interlocate with immune cells and neurons of the enteric nervous system [6]. Probiotics help in bacterial colonization of the intestines, which is crucial for the proper development and growth of the immune and endocrine systems. A probiotic consisting of *Bacillus subtilis* given to chickens prevents complications after exposure to heat stress. In turn, the addition of *Lactobacillus* reduces the population of *Escherichia coli* in the cecum. These are the positive effects of probiotics on heat stress [88]. The cecal microbiota ferments prebiotics in the form of undigested carbohydrates and, as a result, stimulates the production of metabolites, including SCFAs. SCFAs, particularly butyrate, enhance the integrity of the intestinal mucosa by binding to endocrine L-cells [89]. In turn, prebiotics are substances that stimulate the growth of beneficial microorganisms [90].

The most popular are oligosaccharides GOS, mannanoligosaccharides (MOS), fructooligosaccharides (FOS), xylooligosaccharides (XOS), and inulin are fermented by intestinal bacteria, which produce SCFA and lactic acid. Stimulation with prebiotics promotes the growth of the abundance of *Lactobacillus* and *Bifidobacterium* bacteria, which are responsible for inhibiting the growth of pathogenic bacteria in the body [91]. Donalson et al. [92] showed that a diet enriched with 0.75% fructooligosaccharides reduced the occurrence of *Salmonella* spp. in the liver and ovaries. This is due to an increase in the abundance of lactic acid bacteria in the intestines and an increase in intestinal peristalsis [92]. Fowler et al. [93] studied the effect of MOS (250 ppm) on ROSS 308 broilers. They showed that oral administration of this prebiotic from the cell wall of *Saccharomyces cerevisiae* alleviates the effects of heat stress in chickens by increasing the abundance of butyrate-producing bacteria in the intestines. MOS prevents adherence and colonization of the intestine and liver by pathogenic bacteria *E. coli* and *Salmonella* spp. Additionally, MOS increases the height of the intestinal villi. Prebiotics selectively stimulate anti-inflammatory taxa growth and inhibit pro-inflammatory taxa growth [93]. *Lactobacillus* and *Bifidobacterium* bacteria reduce fat accumulation in the liver and minimize serum lipid concentration [94].

### Intestinal dysbiosis

Following infection with bacteria that cause intestinal dysbiosis such as *Escherichia coli*, *Staphylococcus aureus*, *Salmonella enteritidis*, and *Enterococcus faecalis*, liver disease has been noted in hens. These pathogens were shown to cause extracellular amyloid deposition, and such a phenomenon is avian liver amyloid degeneration. A common disease occurring due to dysbiosis is fatty liver in chickens. Liposaccharides produced by *Escherichia coli* are endotoxins derived from the outer membrane of Gram-negative bacteria. They are detected in blood from the portal vein, indicating that the intestinal epithelium absorbs LPS and, if overdosed, can induce liver disease. The inflammatory response induced by LPS causes fatty liver. Dysbiosis is defined as an imbalance between the amount of harmful and defensive intestinal bacteria. It can affect the degree of hepatitis or liver fibrosis [13, 62].

Intestinal dysbiosis is involved in the pathogenesis of autoimmune liver diseases. These include primary biliary cirrhosis (PBC) and primary sclerosing cholangitis (PSC). Both conditions are chronic liver diseases, which development is mediated by the immune system. These dysfunctions are characterized by portal inflammation and slow progression. The causes of these diseases are intestinal dysbiosis, a change in the composition of the intestinal microbiota, a change in the composition of bile acids, unfavorable bacterial products (PAMP), and their metabolites [68]. Cirrhosis of the liver is characterized by the loss of liver cells and irreversible cicatrization. Intestinal dysbiosis in liver cirrhosis is accompanied by impaired intestinal barrier function and pathological distribution of bacteria. Bacterial components and toxins reach the liver through the damaged intestinal barrier, simultaneously accelerating the already present liver damage and increasing the systemic inflammatory response. Prebiotics and probiotics prevent cirrhosis [95]. The use of probiotics reduces bacterial translocations, reduces anti-inflammatory effects, and reduces the release of pro-inflammatory cytokines, including TNF-α [96]. Intestinal dysbiosis has been classified as one of the main factors provoking pathogenesis in the liver, which affects the entire gut-liver-brain axis.

### Heat stress

Stress is the host organism’s physiological and psychological response to the disturbance of homeostasis. The digestive tract responds to various stressors, including heat stress [97]. The combination of too high ambient temperature with high relative humidity results in heat stress. It impairs the growth rate and development of the microbiota [98]. It is one of the main environmental challenges when the balance between the energy produced and the energy released from the body is impaired [99]. In poultry production, heat stress is considered one of the main factors negatively affecting egg and meat production and the general welfare of the flock through changes in intestinal microbiota [97]. Digestive tract organs exposed to stress are more susceptible to diseases. Birds exposed to stress factors, e.g., too high temperature and humidity of the environment, poor ventilation, or too long exposure to sunlight, have disturbed energy homeostasis [100].

Heat stress in chickens increases exposure to pathogenic intestinal bacteria such as *Salmonella* spp. caused by increased intestinal membrane permeability. This microorganism can also be detected in the liver, spleen, and muscles [101]. Exposure to high temperatures limits food intake in broilers. This is associated with changes in the activity of appetite-regulating peptides: anorexigenic peptides of the corticotropin-releasing factor family and orexigenic neuropeptide Y. These peptides act peripherally with the HPA axis [102]. Heat stress reduces food intake in laying hens, reduces laying capacity, and increases animal losses [103]. Under the influence of too high temperature, the intestinal mucosa of chickens is damaged, resulting in limited transport of nutrients [104]. In chickens subjected to heat stress, an increased abundance of *Escherichia coli* and *Clostridium difficile*, which produce alpha toxins that cause necrotizing enterocolitis, was found [105]. Two mechanisms mediate the impact of heat stress on the intestinal epithelium. The first mechanism is the production of reactive oxygen and nitrogen species in response to too high temperatures [106]. The second mechanism involves the production of pro-inflammatory cytokines, which is facilitated by thermal stress [107]. These cytokines include interleukin-2, produced by T cells [108].

The HPA axis is a system that mediates the body’s response to stressors. Its activation releases ACTH and stimulates corticosterone production in birds [109], increasing its level in the blood. Consequently, lower food absorption, reduced immune response, and inflammation development are observed [110]. Corticosterone, cytokines, and selected hormones are factors common to the CNS, the immune system, and the endocrine system. Two catecholamines, epinephrine and norepinephrine, regulate the synthesis of cytokines reducing the expression of pro-inflammatory interleukin-12 and interferon-gamma, and increasing that of regulatory interleukin-10 [14]. The parasympathetic system receives signals from visceral organs and tissues or sends them back to the HPA axis [18]. In studies on chickens, it was shown that heat stress and irritation with *Clostridium* led to a decrease in the concentration of serotonin, epinephrine and norepinephrine in the hypothalamus and dopamine in the midbrain [111]. Dietary supplements (probiotics, prebiotics, and synbiotics) were used to alleviate the effects of heat stress [112]. The intestinal microbiota is sensitive to changing temperatures. A differentiated microbiome is necessary to maintain optimal regulation of signaling pathways in the host organism [113].

Heat stress threatens both humans and animals. Animals have an organism-specific thermal comfort zone necessary for proper functioning physiological functions. When the temperature exceeds the comfort zone’s upper limit, heat stress begins in the animals [114]. The organisms of most animals developed phenotypic reactions (e.g., reduced daily feed intake) in response to heat stress. Unfortunately, a higher temperature is a beneficial factor for the growth and development of pathogens in the host [115]. In defense against the harmful effects of heat stress, animal organisms, including their microbiome, have developed molecular responses to repair damages and protect against their deterioration. Chronic exposure to heat stress reduces the number of type 1 T cells in the body while increasing the number of type 2 T lymphocytes. The imbalance causes changes in cytokine production [116]. Chronic heat stress causes an increase in the abundance of A-8 thermal shock proteins in chickens. These are proteins involved in the immune response [117]. Rapid and extensive transcriptional changes follow the heat shock. Transcription factors activating protein-1 (AP-1), a regulator of transcription and immunity, are activated in the gut to increase the immune response [118]. Heat stress stimulates the sudden and rapid release of the anti-inflammatory cytokines IL-1α, IL-8, and IL-10 into the bloodstream.

Heat stress decreases the expression of zonula occludens 1 in the jejunum and occludin in the ileum of broiler chickens, whereas it increases the expression of IL-1β, IL-6, IFN-γ and TLR4 in both segments of the small intestine. The exposure to heat also contributes to a reduced abundance of *IL-10* mRNA in jejunum and ileum, showing that the impaired balance between pro- and anti-inflammatory cytokines contributes to the disruption of intestinal barrier function [119]. Heat stress lowers the expression of cholecystokinin mRNA in the duodenum and jejunum [120]. Under the influence of heat stress, the intestinal mucosa increases the ability to absorb sugars by up-regulation of GLUT expression. A decrease in GLUT2 expression was observed in the gut in broiler chickens after prolonged exposure to light [121]. The hypothalamic-pituitary axis affects the intestinal microbiota through ASN and the brain [122]. During *Salmonella typhimurium* infection, intestinal neurons produce IL-18, essential for the production of an antimicrobial protein in goblet cells [123]. Prolonged heat stress activates the HPA axis, increasing TNF-β and corticosterone concentration. Corticosterone disrupts the composition of the intestinal microbiota, causing an increase in the permeability of the gastrointestinal tract to pathogenic bacteria [124].

## Summary

Although the concept of the gut-liver-brain axis is relatively new, the number of articles on the subject is small. It can be assumed that with an increase in understanding of the functioning of this relationship in the future, many poultry diseases will be reduced or eliminated. In addition, deepening knowledge and further targeted research will contribute to eliminating losses in animal production, including poultry production worldwide. For animal breeders, the proper growth and development of livestock are crucial. Therefore, it is essential to understand the inter-organ impact to control and eliminate unwanted economic losses through relevant factors.

## Data Availability

Not applicable.

## Abbreviations

ACTH: Adrenocorticotropin hormone
ANS: Autonomic nervous system
AP-1: Transcription factors activating protein-1
CNS: Central nervous system
CRF: Cytokines activate corticotropin-releasing factor
DAMP: Damage-associated molecular patterns
E: Epinephrine
ENS: Enteric nervous system
FOS: Fructooligosaccharides
GABA: Gamma-aminobutyric acid
GLP-1: Glucagon-like peptide-1
GLP-2: Glucagon-like peptide 2
GOS: Galactooligosaccharide
HPA: Hypothalamic-pituitary-adrenal
LPS: Lipopolysaccharides
MOS: Mannanoligosaccharides
MSH: α-melanotropic hormone
NE: Norepinephrine
PAMP: Pathogen-associated molecular patterns
PBC: Primary biliary cirrhosis
POMC: Proopiomelanocortin
PP: Pancreatic polypeptide
PSC: Primary sclerosing cholangitis
PSP: Pathogen-specific patterns
SCFA: Short-chain fatty acid
sIgA: Secretory immunoglobulin A
TLR: Toll-like receptor
XOS: Xylooligosaccharides
5-HIAA: 5-Hydroxyindoleacetic acid
